# Correlations between body postures and musculoskeletal pain in guitar players

**DOI:** 10.1371/journal.pone.0262207

**Published:** 2022-01-04

**Authors:** Sigal Portnoy, Shlomit Cohen, Navah Z. Ratzon

**Affiliations:** Sackler Faculty of Medicine, Department of Occupational Therapy, Tel Aviv University, Tel Aviv, Israel; University of Illinois at Urbana-Champaign, UNITED STATES

## Abstract

**Background:**

The guitar-playing community is the largest group at risk of developing playing related musculoskeletal disorders. A thorough investigation of the relationships between the various risk factors and players’ report on musculoskeletal pain using objective and accurate means of assessment has yet to be reported.

**Purpose:**

(a) to explore the correlations between demographic characteristics, anthropometric measurements, playing habits, and personal habits of guitar player and their complaints of musculoskeletal pain, (b) explore the correlations between the upper body kinematics of guitar players during playing the guitar and their complaints of musculoskeletal pain, and (c) compare the upper body kinematics of guitar players during playing the guitar while sitting versus standing.

**Methods:**

Twenty-five guitar players (27.5±4.6 years old) filled out questionnaires regarding their guitar-playing habits, and the Standardized Nordic Questionnaires for the analysis of musculoskeletal symptoms. Kinematics of their torso and upper limbs were tracked while they played a tune twice, once while sitting and once while standing.

**Results:**

We found moderate correlations between the number of painful joints in the last year and factors, such as physical comfort while playing, years of playing, and position during playing. During standing, lower back pain severity correlated with the rotation range of the torso, while during sitting, it moderately correlated with the average radial-ulnar deviation of the right wrist. During sitting, we found higher anterior and right tilt of the torso, combined with greater abduction of the right shoulder, higher flexion in the left shoulder and higher radial deviation in the left wrist.

**Conclusion:**

Our results point to several risk factors, related both to playing habits but also to playing posture, which should be considered by the guitar players in order to prevent playing-related musculoskeletal disorders.

## 1. Background

Work-related musculoskeletal conditions have been one of the most common health-related issues in recent decades. Work-related musculoskeletal disorders are found in many professions, including professional musicians [1–4]. Music-related musculoskeletal disorder conditions, known as Playing Related Musculoskeletal Disorders (PRMD) [3,5,6], defined as any weakness, numbness, pain, tingling or other symptoms that interfere with the ability to play a musical instrument at the level that the musician is accustomed to playing [3,7].

The prevalence of PRMD among music students ranges from 43% to 63%, and more than 80% among professional musicians [4–6,8,9]. A study that quantified the prevalence and consequences of musculoskeletal symptoms in symphony orchestra musicians [10] showed that women had a significantly higher risk for reporting symptoms compared to men, and the effect of these symptoms lasted for more days The prevalence of PRMD reported over the last year in classical guitar players was 88.9% [11]. Despite the similarity of physical risk factors among all musicians, the risk level for developing PRMD varies among musicians that play different instruments. This level is related to the instruments’ weight and particular specifications related to each instrument, along with the musicians’ posture and body position while playing [4,12,13]. The prevalence of the PRMD is exceptionally high among string players, e.g., violin, guitar, and mandolin [5,12]. In guitarists, two large sample sized studies reported 61.3% pain prevalence for 1 year, as reviewed by [14]. The trunk and upper extremities are usually the most affected areas of injury [2]. The most common diagnoses of PRMD are musculotendinous overuse injuries, the neurological problem of focal dystonia and compressive neuropathy [2].

Health concerns related to musicians and music performers have gained growing attention in recent years with the growth of the music industry. Within the musician population, the rise of the number of guitar players has placed them as the largest population of musicians [15]. As such, the guitar-playing community is the largest group at risk of developing PRMD. The most frequently identified painful anatomical areas were the wrist, used to play chords (fretting hand) (41.8%), back (17.2%), and the strumming hand (13%) [15]. Since there are differences between guitarists in various factors, e.g., number of playing instruments and type of guitar mastered, the effect of these factors on the type and frequency of the PRMD should be explored. A study on various styles of guitarists found that 61.3% of the participants experienced pain related to playing, regardless of the instrument (acoustic guitar, electric guitar, electric bass, or banjo). Also, a recent study showed that the prevalence of musculoskeletal complaints among bassists playing at least two types of instruments (multi-instrumentalists) was similar to that among bassists playing one type of bass instrument (mono-instrumentalists) [16].

Guitar playing is a challenging and arduous process that involves hours of practice and a range of physical abilities, such as unnatural body posture, repetitive arm, wrist and finger movements [15,17]. While playing, guitar players adopt different body postures, depending on the type of guitar they are using. Classical and acoustic guitar players, for example, typically play while sitting. Electric guitar and bass, on the other hand, normally play while standing. To the best of our knowledge, there are no reports comparing biomechanics of playing between sitting and standing postures in guitar players. In brass players, the respiratory mechanics between sitting and standing postures was reported [18] and recommendations for sitting postures were provided [19]. Guitar players’ positioning influences the origin positions of the participating organs, leading to different function of the limbs and back [17]. The vast majority of the guitar players activate extrinsic forearm flexor muscles more than extensor muscles, and thus the flexor muscles endure a more direct exertion of force [20]. The musicians are required to grip the plectrum or the guitar directly with their fingers. They do this with flexed fingers and wrist to reach the strings. The hand playing the chords requires the fingers to produce small, repetitive movements. Moreover, during playing, the wrist position is almost at its maximum range of flexion, a position that reduces the finger force. Some positions also generate static tension over time and create maximum pressure on the tendons and the Median nerve in the carpal tunnel. These postures are often constant and continuous or, at times, repetitive, fast-paced movements. The strumming that induces the vibration of the strings is also considered a risk factor in the physical environment.

Musicians need to maintain the criteria of skills that are no less than those of professional athletes. Nonetheless, unlike athletes, there is inadequate emphasis on the physical and mental conditions required in the playing environment [6]. Only few studies focused on the population of guitar players. Therefore, investigation of the characteristics of guitar players is warranted. Such investigation should aim to understand the relationships between the various risk factors and players’ report on musculoskeletal pain using objective and accurate means of assessment. Due to the scarcity of studies on guitar players and the high prevalence of the PRMD in the guitar-playing population, we aimed to (a) explore the correlations between demographic characteristics, anthropometric measurements, playing habits, and personal habits of guitar player and their complaints of musculoskeletal pain, (b) compare the upper body kinematics of guitar players during playing the guitar while sitting versus standing, and (c) explore the correlations between the upper body kinematics of guitar players during playing the guitar and their complaints of musculoskeletal pain.

## 2. Methods

### 2.1 Participants

We recruited 25 guitar players (22 men, mean age and SD 27.5±4.6 years old, height 175.5±8.0 cm, weight 73.8±18.0 kg, 21 with right hand dominancy). Inclusion criteria were 18–35 year old guitar players, playing for at least 5 years and practice at least 20 hours a week. Exclusion criteria were any medical condition that causes swelling or sensory impairment (e.g. diabetes, arthritis, and pregnancy), excluding diagnoses identified as playing-related musculoskeletal injuries, e.g. De Quervain’s tenosynovitis, Carpal Tunnel Syndrome, tendonitis, and Trigger finger. Guitar players accustomed to a reversed guitar for left-handed individuals were also excluded from the study. We did not exclude guitar players with pain at the day of their examination since the literature shows high prevalence of pain in this population, so that an examination of the subjects, as they are playing in this state, reflects their daily reality. Also, when comparing sitting to standing kinematics, the differences in an intra-subject study design should not affected by this factor. A total of forty guitar players were approached: 2 were excluded for playing a reversed guitar, 4 were excluded for exceeding the age range, and 9 were excluded for not practicing more than 20 hours a week. The study was approved by the Tel Aviv university’s ethics committee.

### 2.2 Tools and protocol

Each subject read and signed an informed consent form pretrial. Then, 19 passive reflective markers were placed on the torso and both upper limbs in anatomical locations (Spinous process C7, top and bottom of the sternum, as well as bilateral placements at the acromion, lateral epicondyle, radial and ulnar styloid joints, 2^nd^ and 5^th^ metacarpophalangeal joints. Four additional markers were placed at mid-lateral forearm and upper arm for error correction during tracking) [21,22]. The subject was situated in a room, equipped with 6 motion capture cameras (Qualysis, Sweden). This method for quantifying upper limb kinematics was found to be reliable and provide good accuracy for tasks such as keyboard typing [22] and guitar playing [23]. After calibration of the system and recording of a static trial, each subject played a familiar 2-minute tune, suitable for the acoustic guitar, provided for this study and data were captured at 100Hz. The same tune was played to all subjects while they were playing, so that all subjects played at identical tempo. The tune was played twice, once while the subject sat on a chair and once while the subject was standing up. This was done in a cross-over design so that half the subjects began playing while sitting and the other half started playing while standing. The subjects then filled out a demographic questionnaire (age, sex, total years of playing, playing hours per day and week, warm up time before playing, durations of breaks during practice). They also filled out the Standardized Nordic Questionnaires (SNQ) [24] for the analysis of musculoskeletal symptoms and rated their health status and their physical comfort while playing on a scale from ‘0’ (extremely bad) to ‘10’ (extremely good).

### 2.3 Post-processing

Thirty seconds out of each 2-minute recording were selected, according to the marker capture quality. Since the selected tune was somewhat repetitive and constant in its tempo, we expected negligible variability in kinematics between any 30s segments taken from the 2-minute recording. The data were then analyzed via a commercial software (Visual 3D, C-motion, MD, USA). The angle of the torso was define according to the coordinate system of the lab and the angles of the shoulder, elbow and wrist joints were defined as the angle of the distal segment in relation to the angle of the proximal segment. For each joint, we computed the mean and range of motion.

Data analyses were performed in SPSS v25. In order to explore the correlations specified in our aims, we conducted the Spearman’s rank correlation test. We performed the Mann-Whitney U test to affirm that there were no statistically significant differences in kinematics between the group who was first tested while sitting and then standing versus the group who was first tested while standing and then sitting. Then, in order to compare the upper body kinematics while sitting versus standing, we performed the Wilcoxon signed-rank test. The effect size, *r*, was calculated using the following equation [25]:

$$r=\frac{Z}{\sqrt{N}}$$ (1)

Statistical significance was set to p<0.05, however we performed the Bonferroni correction, as follows: for the comparison of kinematics between sitting and standing, we divided the p-value by 13 due to the multiple variables, so that the actual statistical significance was set to p<0.0038, and for the correlations we divided the p-value by 8 due to the multiple variables, so that the actual statistical significance was set to p<0.0063.

## 3. Results

The guitar players who participated in this study had between 5 and 25 years of experience (mean and SD of 13.7±5.8 years). Their left and right hand spans were 22.2±1.3cm and 21.3±1.3, respectively. They reported playing between 2 and 7 hours a day (mean and SD of 3.8±1.2 years) and between 20 and 40 hours a week (mean and SD of 23.2±5.7 years). Twenty two subjects (88%) reported sitting during practice and four subjects (16%) reported using an adjusted chair. Ten participants (40%) reported warming up before practice (average and SD of warm up time was 9.4±6.9 minutes) and taking breaks during practice (break duration 9.9±6.1 minutes and the breaks were taken every 70.6±39.6 minutes).

**Fig 1:**
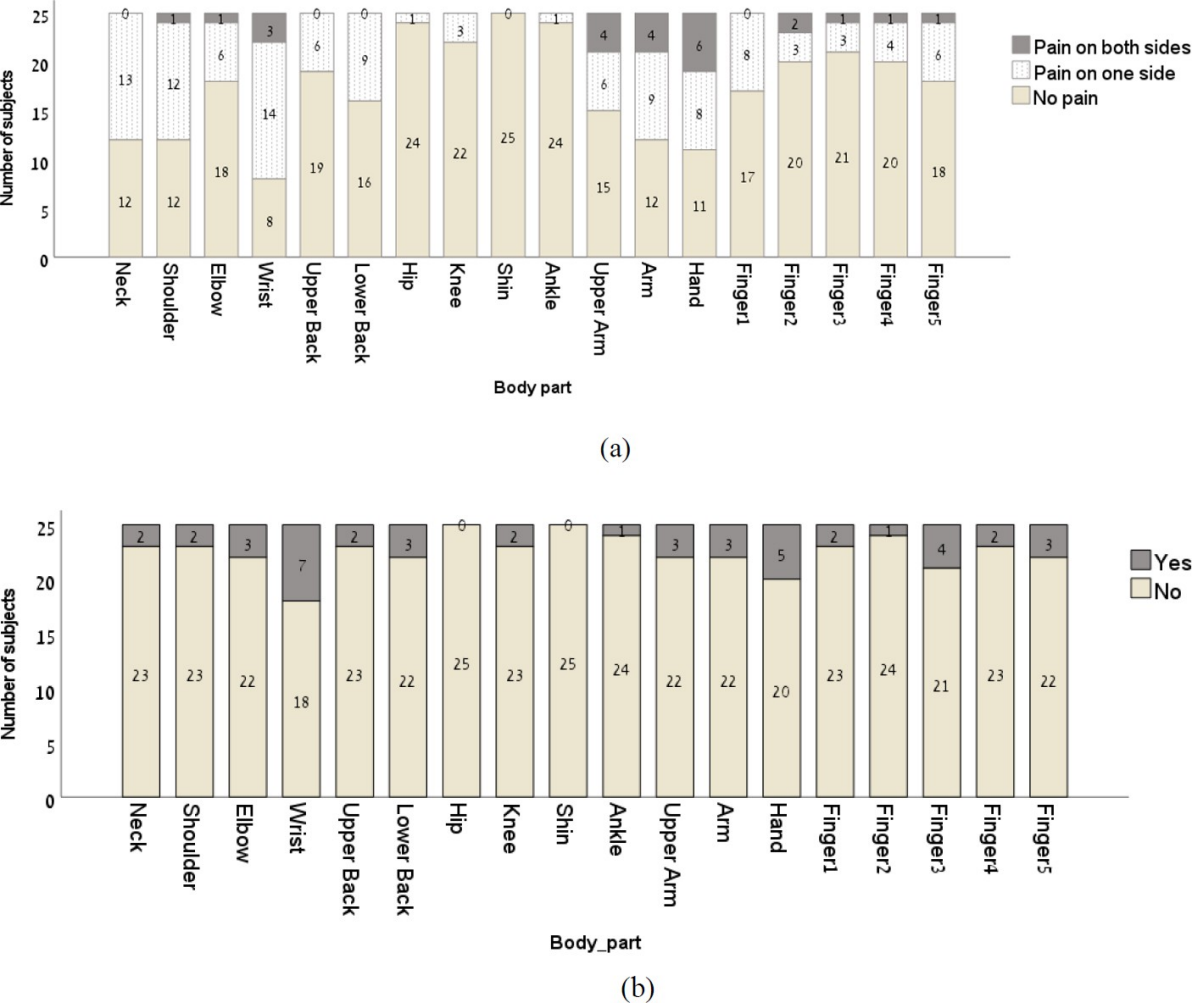
Subjective reports of (a) pain and (b) refraining from performing activity that involves the painful joint in the last year.

From the SNQ, all of the subjects reported having between 1 and 18 painful joints in the last year (average and SD of 6.4±5.0 painful joints; Fig 1A) and none to 8 painful joints in the last week (average and SD of 2.4±2.4 painful joints). Fourteen subjects (56%) did not refrain from work or activity due to joint pain. Of the eleven subjects who reported refraining from performing activity with the painful joint, the number of joints involved were between 1 and 12 (Fig 1B).

The statistically significant correlations found between demographic characteristics, anthropometric measurements, playing habits, and personal habits of guitar player and their complaints of musculoskeletal pain are presented in Table 1. There were significant correlations between the severity of pain in the neck, shoulders and lower back with several factors. We found no correlations between the severity of pain in the upper back, upper arm, elbow, arm, hand, fingers, and lower extremity segments with other collected data so these correlations are not presented in Table 1.

**Table 1:** Spearman’s rank correlations demographic characteristics, anthropometric measurements, playing habits, and personal habits of guitar player and their complaints of musculoskeletal pain.

		Personal factors				Playing-related factors			
		Age	Report of health status	Hours of physical activity per week	Hours of housework per week	Physical comfort while playing	The effect of position during playing	Warm up duration	Years of playing
Number of painful joints	In the last week	.396	-.336	-.424*	-.483*	-.220	-.264	.045	.371
	In the last Year	.593***	-.487*	-.531**	-.515**	-.547***	-.465*	.015	.516**
Severity of symptoms	Neck	.230	-.143	-.190	-.604***	-.252	-.086	-.246	.406*
	Shoulders	.280	-.052	.281	-.147	-.404*	-.186	.855***	.257
	Lower back	.302	-.212	-.156	-.337	-.505*	-.380	.475	.787

*p < .05

**p < .01

***p<0.0063.

The differences of the mean angles of the torso, shoulders, and wrists between sitting and standing positions are presented in Table 2.

**Table 2:** Median and interquartile range of mean joint angles (in degrees) while playing in a sitting and standing positions.

		Sitting	Standing	Z	p	r
Torso	Anterior (+)/Posterior (-) tilt	**52.8(35.8–64.1)**	**19.4 (9.5–25.1)**	**-3.783**	**< .001**	-0.757
	Left (+)/Right (-) lateral tilt	**-8.9 ((-16.7)-(-0.6))**	**-3.1((-11.1)-1.4)**	**-2.371**	**.018** ^*^	-0.474
	Left (+)/Right (-) rotation	-5.2 (-15.3–8.4)	9.9(-6.5–17.8)	-0.782	.434	-0.156
Right shoulder	Flexion (+)/Extension (-)	**79.6(34.3–91.2)**	**55.5(45.5–64.3)**	**-2.000**	**.046** ^*^	-0.400
	Abduction (+)/Adduction (-)	**54.9(39.4–61)**	**31.4(26.7–40.4)**	**-3.943**	**< .001**	-0.789
	External (+)/Internal (-) rotation	-37.4((-93.1)-62.2)	-32.2((-65.1)-14.5)	-1.023	.306	-0.205
Left shoulder	Flexion (+)/Extension (-)	**72.4(62.1–80.3)**	**56.4 (65.1–48.9)**	**-3.702**	**< .001**	-0.740
	Abduction (+)/Adduction (-)	16.1 (1.7–26.6)	11.0(5.8–34.1)	-0.684	.494	-0.137
	External (+)/Internal (-) rotation	-11 ((-114.1)-123.9)	-113.5((-176.2)-138.5)	-0.447	.655	-0.089
Right wrist	Flexion (+)/Extension (-)	-0.6((-5.3)-4.2)	-1.2((-5.1)-3.3)	-1.057	.290	-0.211
	Ulnar (+)/Radial (-) deviation	4.8((-2.1)-8.7)	7.5((-4.1)-10.5)	< .001	1.000	0.000
Left wrist	Flexion (+)/Extension (-)	-19.3((-38.5)-(-13.5))	-25.8 ((-34.5)–(-2.5))	-0.091	.972	-0.018
	Ulnar (+)/Radial (-) deviation	**-11.6((0.9)-(-14.6))**	**-1.6((-6.9)-2.6)**	**-2.677**	**.007** ^*^	-0.535

* Non-significant following the Bonferroni correction.

Spearman’s rank correlation coefficients between kinematic variables and the number of painful joints and pain severity are presented in S1 Table. Several kinematic parameters correlated with the reported number of painful joints in the last week and year. During standing, there were high correlations between the range of rotation of the left shoulder and number of painful joints in the last week and year (r = -.873, p = .010 and r = -.811, p = .027, respectively). Also, the range of torso rotation moderately correlated with the number of painful joints in the last week (r = -.471, p = .023). During sitting, there were moderate correlations between the range of lateral tilt of the torso and number of painful joints in the last week and year (r = .446, p = .029 and r = .498, p = .013, respectively), and between the range of torso rotations and number of painful joints in the last week and year (r = .425, p = .049 and r = .476, p = .025, respectively). Additionally, the average abduction-adduction angle of the right shoulder correlated with the number of painful joints in the last year (r = .480, p = .018).

During standing, there were no correlation between the neck pain severity and the kinematic parameters. Conversely, during sitting, there were moderate correlations between the neck pain severity and the range of flexion-extension and lateral tilt of the torso (r = .481, p = .024 and r = .561, p = .004, respectively), as well as between the neck pain severity and the range of flexion-extension and abduction-adduction angle of the left shoulder (r = .517, p = .014 and r = .432, p = .045, respectively).

During standing, the shoulder pain severity moderately correlated with the rotation range of the right shoulder (r = -.493, p = .023) and the radial-ulnar deviation range of the right wrist (r = -.449, p = .028). During sitting, the shoulder pain severity moderately correlated with the range of anterior-posterior tilt of the torso (r = .424, p = .049) and the flexion-extension range of the right wrist (r = .418, p = .042).

During standing, the lower back pain severity moderately correlated with the rotation range of the torso (r = -.533, p = .009) and the average radial-ulnar deviation of the left wrist (r = -.431, p = .040). During sitting, the lower back pain severity moderately correlated with the average radial-ulnar deviation of the right wrist (r = .451, p = .027).

The maximal extension of the right wrist during sitting negatively correlated with the right hand span (r = -.469, p = .021), i.e., shorter hand span was related to more wrist extension.

## 4. Discussion

The main results of the 3 aims of our study were: (a) the exploration of the correlations between demographic characteristics, anthropometric measurements, playing habits, and personal habits of guitar player and their complaints of musculoskeletal pain mainly yielded statistically significant moderate correlations between the number of painful joints in the last year and factors such as physical comfort while playing, years of playing, and position during playing; (b) the comparison of the upper body kinematics of guitar players during playing the guitar while sitting versus standing yielded higher anterior and right tilt of the torso during sitting, combined with greater abduction of the right shoulder, higher flexion in the left shoulder and higher radial deviation in the left wrist; (c) the exploration of the correlations between the upper body kinematics of guitar players during playing the guitar and their complaints of musculoskeletal pain yielded different correlations for sitting versus standing, e.g., during standing, lower back pain severity moderately correlated with the rotation range of the torso, while during sitting, it moderately correlated with the average radial-ulnar deviation of the right wrist).

Although our study population comprised of young adults, we were able to find a correlation between their age and the number of painful joints reported, as older subjects reported a greater number of painful joints. Moreover, the better the reported health status and adherence to physical activity (also related to the household work question), the less painful joints reported. This finding agrees with previous literature showing that physical activity reduces joint pain. For example, in a meta-analysis concerning the effects of workplace physical activity programs on musculoskeletal pain [26], it was reported that physical activity interventions significantly reduce general musculoskeletal pain, as well as neck and shoulder pain.

As for the playing-related factors, the years of playing the guitar correlated with the number of painful joints, which might suggest that the prolonged exposure to ergonomic risk factors during playing the guitar, might have a cumulative injurious effect on the joints. In the future, this may cause osteoarthritis and other musculoskeletal diseases [27]. Importantly, we found that the subjects who reported maintaining physically comfortable postures during playing, had fewer painful joints. Also, the severity of pain in their shoulders and lower back was lower compared to subjects who did not maintain physically comfortable postures. This finding strengthened the importance of studied of Ergonomy in musicians, since occupational therapy interventions, that proves effective in reducing joint pain, can be applied in order to prevent future injuries and pain. Also, since pain at the upper back was reported as the main influence on the performance of guitar players [11], the proposed intervention may maximize the quality of playing.

The main differences in joint positions between sitting and standing postures mainly amounted to forward leaning during sitting, as the guitar is rested on the legs and is not supported by the back muscles. The sitting posture also meant higher wrist extension for individuals with shorter hand span. In the sitting position, the guitar can be easily placed in a more elevated position compared to playing while standing and also further from the body. Consequently, the right upper arm can lean on the guitar by abducting the right shoulder and the left arm has to reach for the guitar by flexing the left shoulder. These differences between the two postures are supported by our findings, depicted in Table 2. While previous literature compared sitting to standing prolonged activities, e.g., [28], reporting differences in lumbar angles between the postures, none explored these in guitar players via the correlations with PRMD. Our results suggest that in both postures, the torso rotations correlated with the number of painful joints. The torso rotation might reflect the whole spinal mobility. Therefore, the reduced mobility of the trunk might be related to lower back pain, as demonstrated in previous studies [29,30]. However, the correlations between the number of painful joints and the shoulder kinematics differed between the two postures: during standing, the left shoulder rotation correlated with the number of painful joints, while during sitting, the right shoulder abduction angles correlated with the number of painful joints. This is finding might be explained by the muscle demands in each position. While there was higher right shoulder abduction during sitting, the kinematics of the rest of the right limb joints were not different compared to their kinematics during standing. This implies that in order to maintain the desired playing quality, the guitar players managed to produce similar kinematics of their distal joints, while the proximal joints postures were altered according to the sitting or standing posture. This outcome would have required some compensation of the muscles, thereby increasing joint reaction forces that overtime may have produced tissue injury and pain. Since shoulder abduction cause narrowing of the subacromial space, the reported shoulder pain might be caused by shoulder impingement syndrome, which is one of the most common causes of shoulder pain, accounting for 44% to 65% of all shoulder complaints [31]. Future studies might consider recording muscle activity patterns in guitar players during sitting and standing positions in order to gain further insight in the biomechanics of these postures.

Unsurprisingly, there were no correlations between the neck pain severity and the kinematic parameters during standing. This may be explained by the upright position that is usually not accompanied by forward tilt of the head, so that the guitar player is looking ahead in a neutral head position. This posture is natural for the neck, so that the weight of the head does not produce excessive torques on the cervical vertebrae. Conversely, in the sitting position, there were moderate correlations between the neck pain severity and the torso and left shoulder kinematics. In this posture, the guitar player is already leaning forward and will occasionally look at the strings and/or fingers. The weight of the head vector moves farther away from the neck, producing higher torques and therefore greater loads on the cervical vertebrae.

The limitations of this study include a relatively small sample size, so that our findings might not relate to the entire guitar playing population. Additionally, since the majority of our study population were men (88%), the report of number of painful joints might have been underestimated compared to a population with equal number of men and women. Also, the measurements were taken in a laboratory settings and the markers, placed on the skin of the subjects, might have altered their movements. We therefore allowed a brief warm-up with the markers so that the subjects will get used to movement with the markers. Also, some of chords might have been more challenging to play, thereby affecting the kinematics. Finally, we did not measure other possible risk factors, e.g. sleeping habits, diet, computer use, and mental anxiety. Additionally, we did not acquire data regarding the width of the guitar strap used by the subjects, which might be relevant to PRMD since it was suggested that a wide strap may decrease the tension on the left trapezius and periscapular muscles [32].

In conclusion, our results point to several risk factors, related both to playing habits but also to playing posture, which should be considered by the guitar players in order to prevent PRMDs. The results of this study may encourage clinicians to build a primary prevention program for guitar players. Ergonomic redesign of the tasks (playing in standing position) and improving personal habits (like physical exercises) might reduce biomechanical strain and assist players by reducing physical demands (range of motion) and increasing their resistance to musculoskeletal injury.

## Supporting information

S1 Table Spearman’s rank correlation coefficients between kinematic variables and the number of painful joints and pain intensity. (DOCX)

S1 Data SPSS file with the data of this study. (SAV)
