# Supplementary material for: Correlations between body postures and musculoskeletal pain in guitar players
Source: PLoS One. 2022 Jan 4;17(1):e0262207. doi: 10.1371/journal.pone.0262207 (PMC8726467; doi:10.1371/journal.pone.0262207)
Supplement: S1 Table — (DOCX) [file pone.0262207.s001.docx]

**Table S1**: Spearman’s rank correlation coefficients between kinematic variables and the number of painful joints and pain intensity.

|  |  | Sitting | | | | | Standing | | | | |
| --- | --- | --- | --- | --- | --- | --- | --- | --- | --- | --- | --- |
|  |  | Number of painful joints | | Pain severity | | | Number of painful joints | | Pain severity | | |
|  |  | In the last week | In the last year | Neck | Shoulder | Lower back | In the last week | In the last year | Neck | Shoulder | Lower back |
| Torso | Range of rotation | .425^*^ | .476^*^ | .304 | .162 | .279 | -.471^*^ | -.355 | .035 | .253 | -.533^**^ |
|  | Range of lateral tilt | .446^*^ | .498^*^ | .561^***^ | .331 | .084 | .091 | .295 | .292 | -.073 | -.312 |
|  | Range of anterior-posterior tilt | .302 | .288 | .481^*^ | .424^*^ | .066 | -.332 | -.191 | .190 | .354 | -.196 |
| Left shoulder | Range of rotation | ¥ | ¥ | ¥ | ¥ | ¥ | -.873^*^ | -.811^*^ | -.334 | -.571 | -.334 |
|  | Range of flexion-extension | .037 | .015 | .517^*^ | .404 | -.240 | -.113 | -.143 | .234 | .301 | -.249 |
|  | Range of abduction-adduction | .194 | .136 | .432^*^ | .280 | .157 | .219 | .086 | .262 | .298 | .129 |
| Right shoulder | Range of rotation | -.157 | .025 | .100 | .437 | -.284 | .098 | .200 | .272 | -.493^*^ | .188 |
|  | Average abduction-adduction angle | .281 | .480^*^ | .059 | -.348 | .147 | .109 | .220 | -.217 | -.312 | -.031 |
| Right wrist | Range of flexion-extension | .129 | .218 | .144 | .418^*^ | -.045 | -.003 | -.085 | .134 | -.171 | -.259 |
|  | Range of radial-ulnar deviation | .065 | -.043 | -.015 | .295 | -.031 | -.078 | -.183 | -.110 | -.449^*^ | -.305 |
|  | Average radial-ulnar deviation | .118 | -.074 | .273 | .332 | .451^*^ | .087 | .143 | .283 | .311 | .019 |
| Left wrist | Average radial-ulnar deviation | .122 | .070 | -.103 | .077 | -.083 | -.400 | -.179 | -.119 | -.025 | -.431^*^ |
| ^*^p<.05, ^**^p<.01, ^***^p<0.0063  ¥ Data were unavailable due to technical issues. | | | | | | | | | | | |
